# Adoption of Biosimilar Infliximab for Rheumatoid Arthritis, Ankylosing Spondylitis, and Inflammatory Bowel Diseases in the EU5: A Budget Impact Analysis Using a Delphi Panel

**DOI:** 10.3389/fphar.2017.00322

**Published:** 2017-05-31

**Authors:** Tim A. Kanters, Jelena Stevanovic, Isabelle Huys, Arnold G. Vulto, Steven Simoens

**Affiliations:** ^1^Institute for Medical Technology Assessment, Institute of Health Policy and Management, Erasmus University RotterdamRotterdam, Netherlands; ^2^Department of Pharmaceutical and Pharmacological Sciences, KU LeuvenLeuven, Belgium; ^3^Hospital Pharmacy, Erasmus MC, University Medical Center, RotterdamRotterdam, Netherlands

**Keywords:** budget impact, biosimilars, infliximab, rheumatoid arthritis, ankylosing spondylitis, inflammatory bowel disease

## Abstract

**Introduction:** Introducing biosimilar infliximab for the treatment in rheumatology (rheumatoid arthritis and ankylosing spondylitis) and inflammatory bowel disease (Crohn's disease and ulcerative colitis) may reduce treatment costs associated with biologics. This study aimed to investigate the budget impact of adopting biosimilar infliximab in five European countries, considering that the budget impact includes the adoption of biosimilar infliximab and the availability of biologic alternatives such as vedolizumab, biosimilar etanercept, biosimilar rituximab, and other relevant factors.

**Methods:** An existing budget impact model was adapted to forecast the budget impact in the UK, Germany, France, Spain, and Italy. Epidemiological parameters were derived from published literature reviewed in July 2015. Current market shares of biologics were derived from Therapy Watch (2012/2013 data). Respondents in a Delphi panel, conducted in 2015 and consisting of several leading rheumatologists and gastroenterologists from different nationalities, were asked to forecast uptake of biosimilar infliximab and estimate the proportion of patients eligible for a particular type of biological treatment, including biosimilar infliximab. Scenario analyses assessed the influence of various factors, including price reductions, on the budget.

**Results:** Uptake of biosimilar infliximab was particularly expected for naïve patients; switching patients that already received other biologics was not expected much. Market shares after 5 years of biosimilar infliximab were ~2% in rheumatology in all five countries and in gastroenterology ranged from 4% in France to over 30% in Italy. Except for France, budgets were expected to decrease for rheumatologic diseases. For gastroenterology, budgets were expected to decrease in Spain and Italy. Budgets were expected to increase substantially in the UK and Germany, due to the introduction of vedolizumab in the studied period. In France, budget was expected to slightly increase for ankylosing spondylitis, Crohn's Disease, and ulcerative collitis. Savings in budget were expected in all countries, for all diseases, when larger price discounts on biosimilar infliximab were used.

**Discussion and Conclusion:** This study has shown that only when price reductions are large enough (i.e., 50% or more), physicians indicated that they will prescribe biosimilars. Policy makers should ensure substantial price reductions and stimulate physicians to use biosimilar products, to obtain savings in healthcare budgets.

## Introduction

Biological drugs acting as anti-tumor necrosis factors (TNF), inhibitors of the T- and B-cell costimulation, and inhibitors of the interleukin (IL)-6 and the IL-1 have been shown to successfully reach treatment targets (e.g., remission, low disease activity) and improve patients' quality of life for several gastrointestinal and rheumatic diseases in a number of clinical situations (Mathias et al., 2000; Dignass et al., 2010, 2012; Lichtiger et al., 2010; Braun et al., 2011; Smolen et al., 2014). Both knowledge and experience gained with these drugs have supported their acceptance by gastroenterologists and rheumatologists, and the drugs' integration in the treatment algorithms of inflammatory bowel disease (IBD), including Crohn's disease (CD) and ulcerative colitis (UC)—together also named gastroenterological indications, rheumatoid arthritis (RA), and ankylosing spondylitis (AS)—together also named rheumatological indications (Dignass et al., 2010, 2012; Braun et al., 2011; Smolen et al., 2014).

Treatment with biological drugs in RA, AS, CD, and UC is considered highly effective, but often comes at a high cost. In the European Union Five (EU5) markets including France, Germany, Italy, Spain, and the United Kingdom, annual biological drug acquisition costs in RA range from €6,650 per patient (B cell costimulation inhibitor rituximab in Italy) to €23,395 per patient (T cell costimulation inhibitor abatacept in Germany) assuming list prices and use at the label dose (Italian hospital pharmacist, 2012; Rote Liste, 2013). Notably, acquisition cost of a specific biological drug may vary across indications due to different dosing regimens, but it always outweighs the cost of conventional non-biologic treatment. Several cost-of-illness studies for RA found that the cost of anti-TNF drugs comprised more than double the total medication expenditure, and was the main contributor to the healthcare outpatient costs (Michaud et al., 2003; Fautrel et al., 2005; Verstappen et al., 2005; Li et al., 2006). Likewise, a cost study in the UK also showed the increased medication costs as a proportion of total costs in AS (Cooksey et al., 2015). Similarly, in a cost study of IBD in the Dutch setting, the cost of anti-TNF drugs was estimated to account for 64 and 54% of the total healthcare costs of CD and UC, respectively (van der Valk et al., 2014).

The recent introduction of biosimilar infliximab (marketed as Inflectra® and Remsima®), an anti-TNF biologic indicated for RA, AS, CD, and UC among other conditions, to the EU markets, may address this issue of increasing financial impact of treatment with biologics. Namely, biosimilar infliximab gained market authorization by the European Medicines Agency (EMA) for the same indications as the reference biologic, but is available at a (up to 25%) lower price in the EU5. However, various factors may influence the adoption of biosimilar infliximab by various stakeholders (e.g., clinicians, payers, patients) and, therefore, its possible impact on healthcare budgets (Moors, 2007). Local costs of biologics, reimbursement policies and budgets, differences in medical practices are some of the factors that may influence patient's access to biological drugs, both brands and biosimilars, and their adoption (Kobelt and Kasteng, 2009; Hockley et al., 2012; Rencz et al., 2015). For clinicians to integrate biosimilar infliximab in treatment algorithms, factors such as the availability of clinical trial evidence and comparability with the reference drug may be of key importance. In particular, biosimilar infliximab was authorized by EMA based on evidence from extensive comparability exercises in quality, safety and effectiveness characteristics to the reference drug, and evidence from clinical trials in patients with AS (PLANETAS) and in patients with RA (PLANETRA). Thus, the adoption of biosimilar infliximab for use in extrapolated indications, CD and UC, may be particularly limited, judging by the initial recommendations proposed by the European Crohn's and Colitis Organization in 2013 (ECCO; Danese and Gomollon, 2013). Their recommendations urge for clinical evidence in patients with CD and UC, and discourage switching from an established biologic to a biosimilar for reasons other than efficacy and safety. Furthermore, the adoption of biosimilar infliximab for RA, AS, CD, and UC at European markets, may be additionally challenged by future dynamics in biological drugs markets involving the availability of a new biological brand for the treatment of IBD, vedolizumab, and possible future introductions of biosimilars of etanercept and rituximab indicated for RA and AS.

The aim of this study is to calculate the impact of adopting biosimilar infliximab for the treatment of RA, AS, CD, and UC on the healthcare budgets in the EU5, taking into account that the budgetary impact is not limited to the adoption of biosimilar infliximab, but also needs to reflect the availability of biologic alternatives such as vedolizumab, biosimilar etanercept and biosimilar rituximab, and other relevant factors.

## Materials and methods

A budget impact analysis (BIA) was performed to compare the “world with biosimilar infliximab” to the “world without biosimilar infliximab” for treatment of RA, AS, CD, and UC in the EU5. The budget impact of treatment with biosimilar infliximab was assessed each year up to 5 years, from the perspective of a healthcare payer (so not including costs in other sectors e.g., productivity costs).

### Adoption of biosimilar infliximab (future market shares)—delphi survey

A two-round Delphi survey was conducted with rheumatology and gastroenterology experts from the EU5 to establish some of the factors influencing adoption of biosimilar infliximab and the corresponding parameter estimates for market shares of biosimilar infliximab for treatment of RA, AS, CD, and UC in the next 5 years.

Clinicians were considered eligible for our study if they: (1) were appraised as local/international experts in their field, (2) are actively involved in treating or supervising the treatment of patients with RA and AS/CD and UC. Additionally, clinicians involved in forming national/international treatment guidelines were preferred. To identify potential participants, MEDLINE was searched for recent publications in rheumatology and gastroenterology concerning treatment recommendations on biologics use in RA, AS, CD, and UC. Additionally, medical directors of Hospira were asked to nominate local clinical experts.

An information letter was sent to physicians setting out the aims of the study and explaining the nature of their contribution. No informed consent from physicians was needed as data were collected and analyzed anonymously. Physicians received a fee for participation in the study. Given that the Delphi survey did not collect health data and did not gather data from patients, no approval from an ethics committee was required as set out in the Belgian law of 7th May 2004.

A total of 106 rheumatology and gastroenterology experts from the EU5 were invited to participate. Each expert received a field—(i.e., rheumatology and gastroenterology) and country—specific survey. In the first survey, participants were asked to reflect in their answers a general attitude and expectations of clinicians in their own country for the adoption of Infliximab biosimilars. Importantly, data from gastroenterology surveys were pooled per each country, while all the response data from the rheumatology field surveys were pooled together because fewer rheumatologists participated in the survey.

In the second round, respondents from the first round received personalized surveys that contained their initial answer and summarized responses from the first round. Here, experts were given the opportunity to change their initial answer in light of other experts' responses.

The surveys examined a number of medical practice related issues that may aid in explaining the expected adoption of biosimilar infliximab, in particular: (1) the number of patients in their institution that are eligible and receiving biologic drugs, (2) reasons for limited access to biological drugs, (3) the role of patients' preferences in biologic treatment choice and rank order of biologics, (4) the general attitude of rheumatologists and gastroenterologists toward the use of biosimilar infliximab in RA, AS, CD, and UC, (5) the expectation of any physician incentives (e.g., prescription quotas, prescription conditions/guidelines, official recommendations on switching, etc.) to prescribe biosimilars infliximab.

The general attitude of rheumatologists and gastroenterologists toward the use of biosimilar Infliximab in RA, AS, CD, and UC, was assessed by comparing the level of comparability of the efficacy and safety profile of biosimilar Infliximab to the one of the reference biologic. Here, levels of comparability considerations were measured on a 5-point Likert scale, ranging from “1” (considered not comparable) to “5” (considered comparable). To indicate the patients' preferences in biologic treatment choice, experts were asked to rank the available drugs from the most to least likely to be chosen by patient. Other survey questions included (e.g., on the future market shares of biosimilar infliximab) were designed to capture estimates on scale 0–100% and were generally stratified by indication (i.e., RA, AS, CD, and UC) and previous use of biological drugs (i.e., use in naïve and existing patients).

### Model structure

An unpublished budget impact model as developed by WG Consulting Healthcare Limited for Hospira was modified and updated to include all currently available biological drugs in the EU5 for treatment of RA, AS, CD, and UC. This model is informed with country-specific epidemiological data, current and future market shares of each biological drug, and direct treatment cost data (see further for data sources). Patient population in the model comprises all patients who are eligible and receive biological drugs for RA, AS, CD, and UC in practice (see further for data sources). Both biologic naïve and existing patients were included.

In the base-case analysis, in the “world with biosimilar infliximab” scenario, existing patients who enter the model may stay on their initial biologic therapy or switch to biosimilar infliximab, while naïve patients may be allocated to any of the already available biological drugs including biosimilar infliximab. For treatment of AS, biological drugs considered were: etanercept, adalimumab, golimumab, infliximab. Next to the aforementioned biologics, treatment of RA also considered certolizumab, rituximab, abatacept, and tocilizumab. For treatment of CD, adalimumab and infliximab were considered, while the treatment of UC considered golimumab, adalimumab, and infliximab. Importantly, next to biosimilar infliximab, a novel treatment alternative considered for treatment of CD and UC was a new biological brand vedolizumab authorized by EMA in 2014. At the time of conducting this study, vedolizumab has only been available in the UK and Germany. Thus, in those two markets, both existing and naïve CD and UC patients who enter the model could receive vedolizumab as a treatment alternative in the base case. After each model cycle, existing patients were assumed to continue treatment with the biological drug assigned in the previous model cycle.

In the “world without biosimilar infliximab” scenario, both naïve and existing patients who enter the model were allocated to currently available biological drugs according to their market shares. In our study, treatment discontinuation and all-cause mortality were neglected. The model provided yearly budget impact estimates. The time horizon of the model was 5 years. The model was developed in MS Excel.

### Epidemiological data

Data on country-specific incidence and prevalence rates were derived from the published literature. We reviewed the literature for relevant publications up until July 2015. Data on the percentage of patients who are eligible and receive biological drugs in practice were based on the experts' opinion assessed in our Delphi survey. The number of patients in each yearly model cycle was adjusted for the country-specific population growth rate.

### Current market shares of biological drugs

Current market shares of biological drugs available in the EU5 for RA, AS, CD, and UC were based on data obtained from Therapy Watch, Research Partnership Ltd (2012 and 2013 data; https://www.researchpartnership.com/what-we-do/syndicated/therapywatch/). Therapy Watch presents an online patient tracking tool that collects real-time information from a panel of specialists recruited to enter online patient record forms via a secure physician website.

### Cost data

Three types of costs directly related to treatment were included in this BIA: annual acquisition costs, administration costs, and therapy monitoring costs. To calculate the annual acquisition costs, official national list prices and use at the label dose were assumed. Price discounts on biosimilar infliximab compared to reference infliximab varied between countries and were (in 2015) 10% in the UK, 14% in Germany, 0% in France, 18% in Spain, and 25% in Italy. These discounts were based on list prices of drugs as provided by national agencies at the moment of data search. The annual acquisition costs assumed no vial sharing. Finally, a distinction was made between the costs occurring in the first year of initiating biologic treatment (induction treatment) and subsequent years (maintenance treatment), as for some treatments (e.g., infliximab, abatacept) dosing differs between induction treatment and maintenance treatment.

For calculating the annual costs of administration and treatment monitoring, national tariffs or cost and recourse use data collected from published literature were used. All costs were inflated to 2014 levels using the harmonized index for consumer product (HICP) for the health sector separately for each country^1^. For comparability, costs for the UK were expressed in Euro values, using a exchange rate of £1 = €1.36 (http://www.xe.com/currencyconverter/convert/?From=GBP&To=EUR; 06/09/2015). Costs were not discounted as recommended by the International Society for Pharmacoeconomics and Outcomes Research for BIAs (ISPOR) Task Force (Mauskopf et al., 2007).

### Scenario analyses

To examine the impact of factors influencing adoption of biosimilar infliximab on the budget, six scenario analyses were conducted. The first scenario assumed biosimilar infliximab would be available at a discount of 50% compared to the reference biologic. The second scenario assumed the availability of biosimilar infliximab at a discount of 75% compared to the reference biologic. The third scenario assumed the availability of clinical trial/observational study evidence confirming the efficacy and safety of biosimilar infliximab in extrapolated indications, CD, and UC. The fourth scenario examined the budget impact of adoption of biosimilar infliximab in Italy, Spain, and France, conditional on the availability of a new biological brand vedolizumab for treatment of CD and UC. Here, the price of vedolizumab was assumed to be the average of prices in the UK (i.e., £2,050) and Germany (i.e., €4,305.60). Similarly, scenarios 5 and 6 examined the budget impact of adoption of biosimilar infliximab conditional on the future availability of biosimilar etanercept for treatment of RA and AS, and biosimilar rituximab for treatment of RA, respectively. In the EU5 markets, biosimilar etanercept and rituximab were assumed to be available at the same discounts compared to their reference drugs as currently is established for biosimilar infliximab. Here, after the first year (from year 2016 onwards), existing patients could switch from their initially assigned biologic therapy to biosimilar etanercept (scenario 5) or biosimilar rituximab (scenario 6) while naïve patients may be allocated to any of the already available biological drugs including biosimilar etanercept (or rituximab) and biosimilar infliximab.

## Results

### Delphi survey

A total of 106 rheumatology and gastroenterology experts from the EU5 were invited to participate in our Delphi survey. Thirty-five (response rate 33%) and seventeen (response rate 17%) experts responded to the first and second round of survey, respectively. Respondent demographics are displayed in Table 1.

**Table 1:** Respondent demographics (round 1) *n* = 35.

	**Gastroenterology experts**	**Rheumatology experts**
Country of residence		
UK	4	1
Germany	5	2
France	3	3
Spain	8	1
Italy	5	3
Working setting	University hospital = 23	University hospital = 7
	Specialized hospital = 1	Specialized hospital = 1
	Regional hospital = 1	University and regional hospital = 1
		Specialized and regional = 1
Years in clinical practice	Mean = 15, range 4–32	Mean = 22, range 12–35
Number of treated patients/whose treatment is supervised per month	Mean = 185, range 15–1,500	Mean = 146, range 25–300
Disease severity of treated patients	Moderate = 28%	Moderate = 40%
	Severe = 19%	Severe = 27%

Respondents believed that efficacy of biosimilar infliximab was comparable to reference infliximab in both rheumatology and gastroenterology; on a scale of 1 (not comparable) to 5 (comparable) median responses were 4 or 5 for all countries in both rheumatology and gastroenterology. Likewise, they believed that safety profile was comparable. Comorbidities were most often mentioned as a reason not to use biologics in treating RA and AS patients that were considered eligible for treatment with biologics. Patient characteristics and patient preferences were other reasons for not using biologics. In CD and UC, patient preferences were most often the reason not to use biologics. In these patients, comorbidities were another reasons not to use biologics. Table 2 provides the development of the number of patients that were considered eligible for treatment with biologics in the five countries over the study period.

**Table 2:** Number of patients eligible for treatment with biologics.

	**Year 0**	**Year 1**	**Year 2**	**Year 3**	**Year 4**	**Year 5**
**RHEUMATOLOGY**						
UK	63,270	63,694	64,101	64,491	64,867	65,227
Germany	62,899	63,050	63,195	63,334	63,468	63,596
France	34,874	35,058	35,238	35,411	35,580	35,743
Spain	45,735	45,625	45,519	45,415	45,315	45,217
Italy	60,698	60,995	61,286	61,571	61,850	62,122
**GASTROENTEROLOGY**						
UK	63,271	63,695	64,106	64,504	64,889	65,263
Germany	27,060	27,125	27,182	27,233	27,279	27,319
France	35,896	36,087	36,267	36,437	36,597	36,749
Spain	11,251	11,224	11,198	11,174	11,151	11,129
Italy	4,079	4,099	4,117	4,133	4,147	4,160

### Market shares

**Figure 1:**
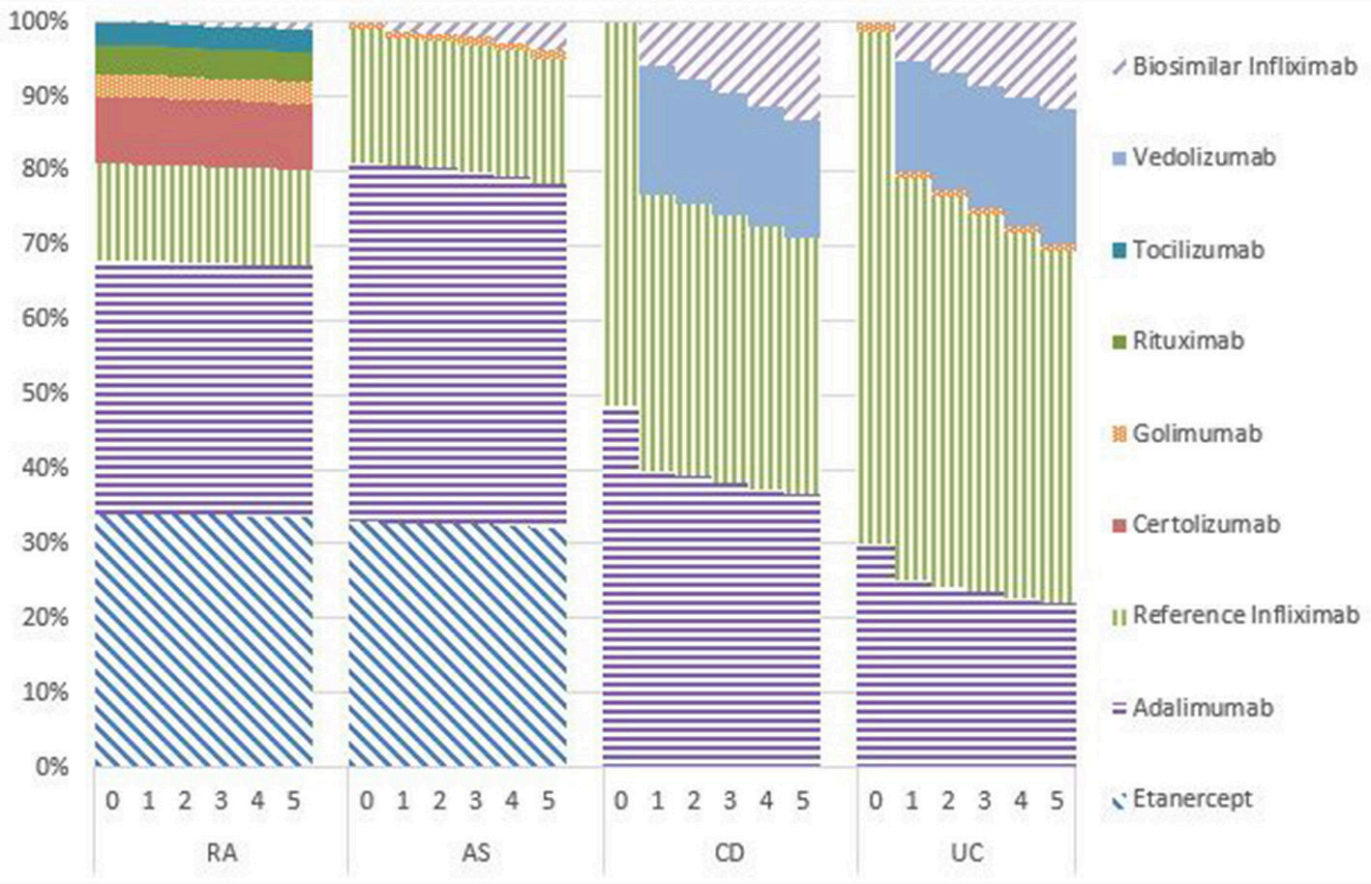
Market shares biologics UK in years 0–5.

**Figure 2:**
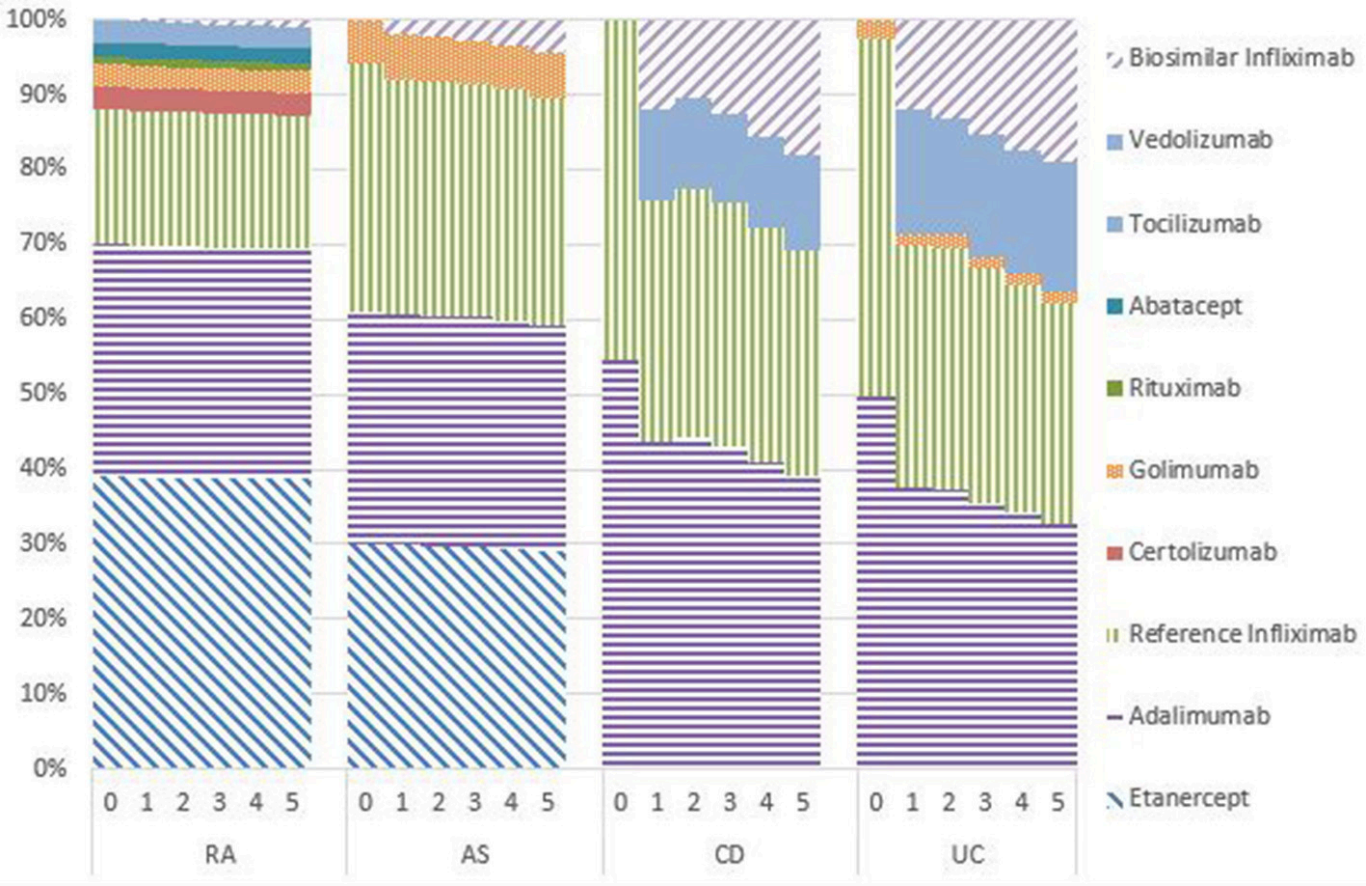
Market shares biologics Germany in years 0–5.

**Figure 3:**
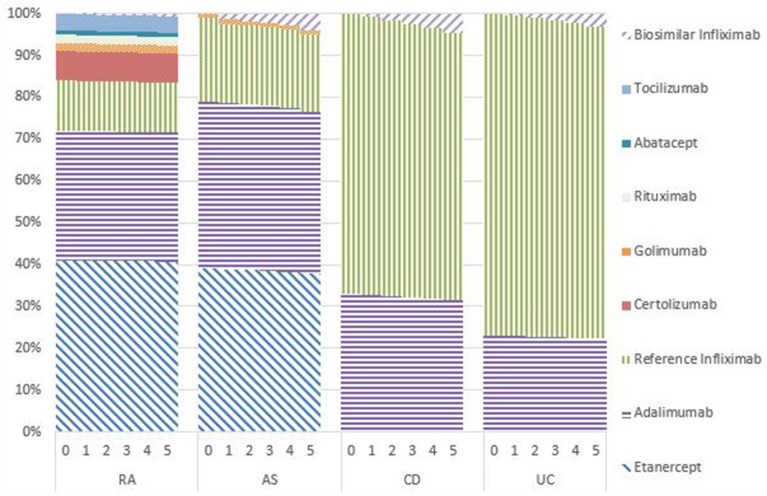
Market shares biologics France in years 0–5.

**Figure 4:**
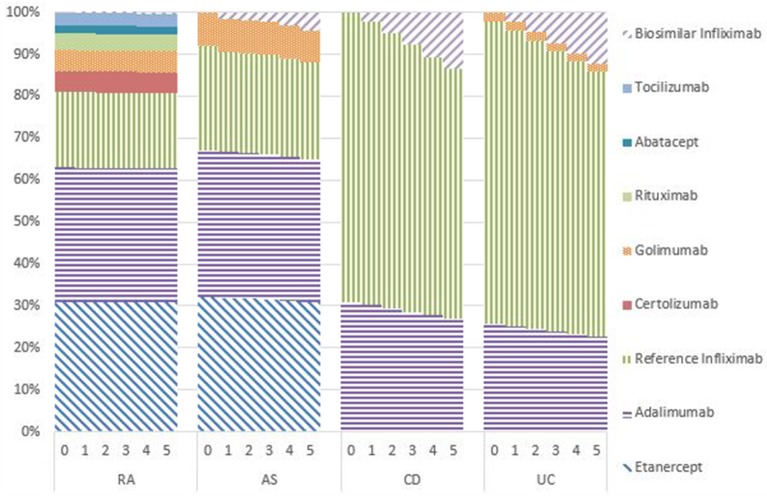
Market shares biologics Spain in years 0–5.

**Figure 5:**
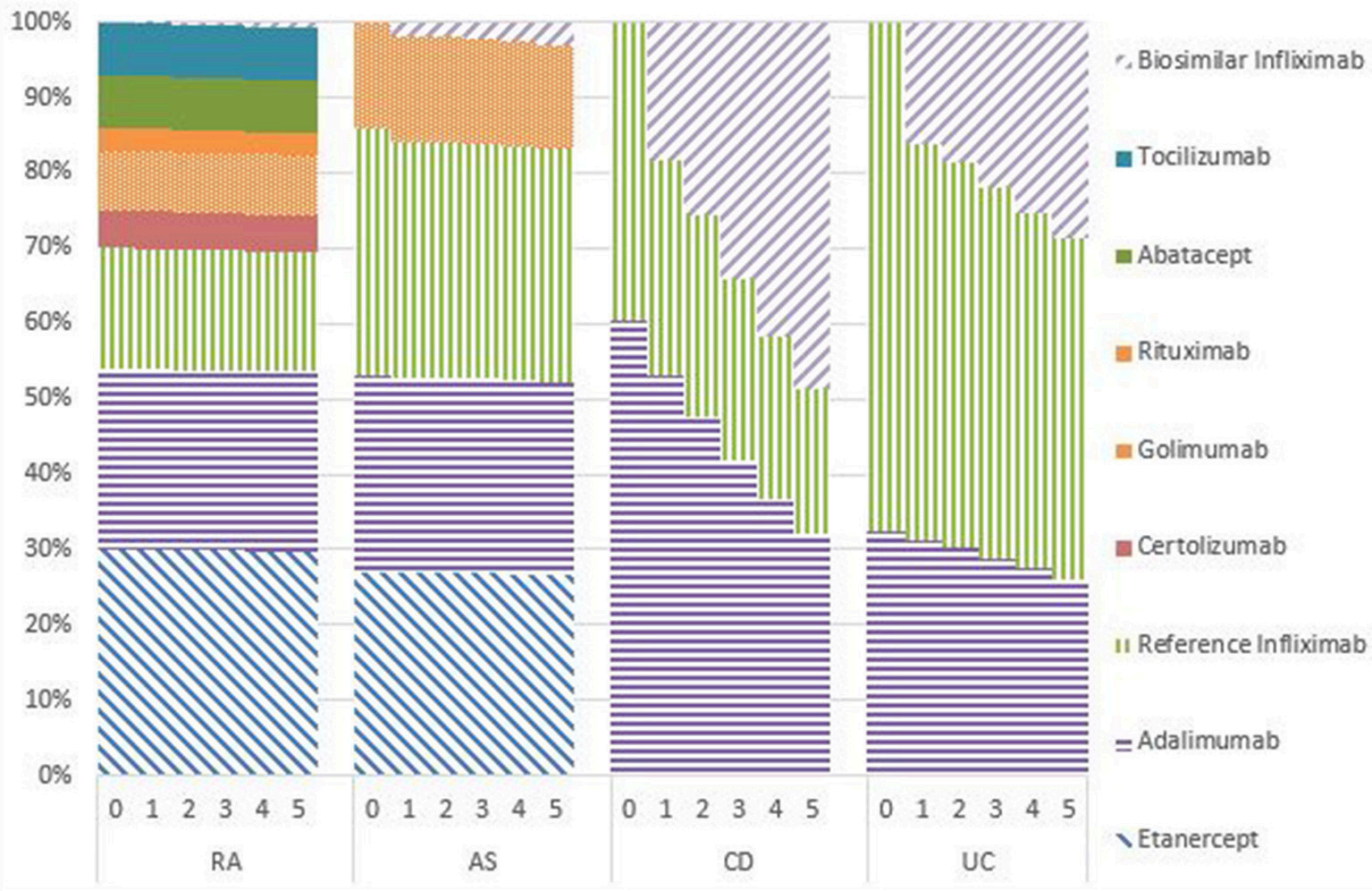
Market shares biologics Italy in years 0–5.

Figures 1–5 provide the market shares of the various biologics over the studied period in the EU5. For all countries, the uptake of biosimilar infliximab was smaller for RA and AS than for CD and UC. In all countries and for all four diseases, the market share of biosimilar infliximab increased over time. The increasing market share of biosimilar infliximab is also shown in Table 3. For RA, physicians in all five countries indicated that 0% of existing patients would switch to biosimilar infliximab. Only 5% of existing AS patients would switch from reference infliximab to biosimilar infliximab. For existing CD (EU5 average 9.5%) and UC (EU5 average 8.7) patients, the share was higher, but still limited. These rates varied substantially between countries, ranging from 0% in France and Spain to 20% in Italy. On average, 2% of CD and UC patients treated with adalimumab would switch to biosimilar infliximab, and 2% of UC patients would switch from golimumab to biosimilar infliximab. Figures 1, 2 further show that a large proportion of CD and UC patients would switch to vedoluzimab in UK and Germany, in addition to the patients that were treated with biosimilar infliximab.

**Table 3:** Proportion of patients receiving biosimilar infliximab (year 0 = 2015).

	**Year 0**	**Year 1**	**Year 2**	**Year 3**	**Year 4**	**Year 5**
**PROPORTION OF TOTAL PATIENTS RECEIVING BIOSIMILAR INFLIXIMAB**						
**Rheumatology**						
UK (%)	0.0	0.5	0.8	1.0	1.4	1.8
Germany (%)	0.0	0.7	1.0	1.2	1.5	1.9
France (%)	0.0	0.8	1.0	1.3	1.7	2.3
Spain (%)	0.0	0.7	0.9	1.0	1.4	1.8
Italy (%)	0.0	1.2	1.4	1.5	1.8	2.2
**Gastroenterology**						
UK (%)	0.0	5.7	7.4	9.2	11.0	12.7
Germany (%)	0.0	11.9	11.2	13.4	16.0	18.4
France (%)	0.0	0.6	1.5	2.3	3.0	4.0
Spain (%)	0.0	2.3	5.0	7.7	10.4	13.1
Italy (%)	0.0	16.8	20.2	24.5	28.7	32.9
**PROPORTION OF NAÏVE PATIENTS RECEIVING BIOSIMILAR INFLIXIMAB**						
**Rheumatology**						
UK (%)	0.0	7.5	7.5	7.0	12.5	16.3
Germany (%)	0.0	7.5	7.5	7.0	12.5	16.3
France (%)	0.0	7.5	7.5	7.0	12.5	16.3
Spain (%)	0.0	7.5	7.5	7.0	12.5	16.3
Italy (%)	0.0	7.5	7.5	7.0	12.5	16.3
**Gastroenterology**						
UK (%)	0.0	30.0	51.3	53.8	56.3	56.3
Germany (%)	0.0	10.0	10.0	30.0	35.0	35.0
France (%)	0.0	10.0	15.0	15.0	15.0	20.0
Spain (%)	0.0	50.0	60.0	62.5	65.0	67.5
Italy (%)	0.0	50.0	47.5	60.0	65.0	70.0

For all four diseases studied, increasing market shares of biosimilar infliximab was mainly driven by naïve patients, which is shown in the lower part of Table 3. For RA, an increasing share of naïve patients would be prescribed biosimilar infliximab over time, reaching 16% in year 5. In the UK, Italy and Spain, more than half of the naïve IBD patients would be treated with biosimilar infliximab from year 3 onwards.

### Budget impact

**Figure 6:**
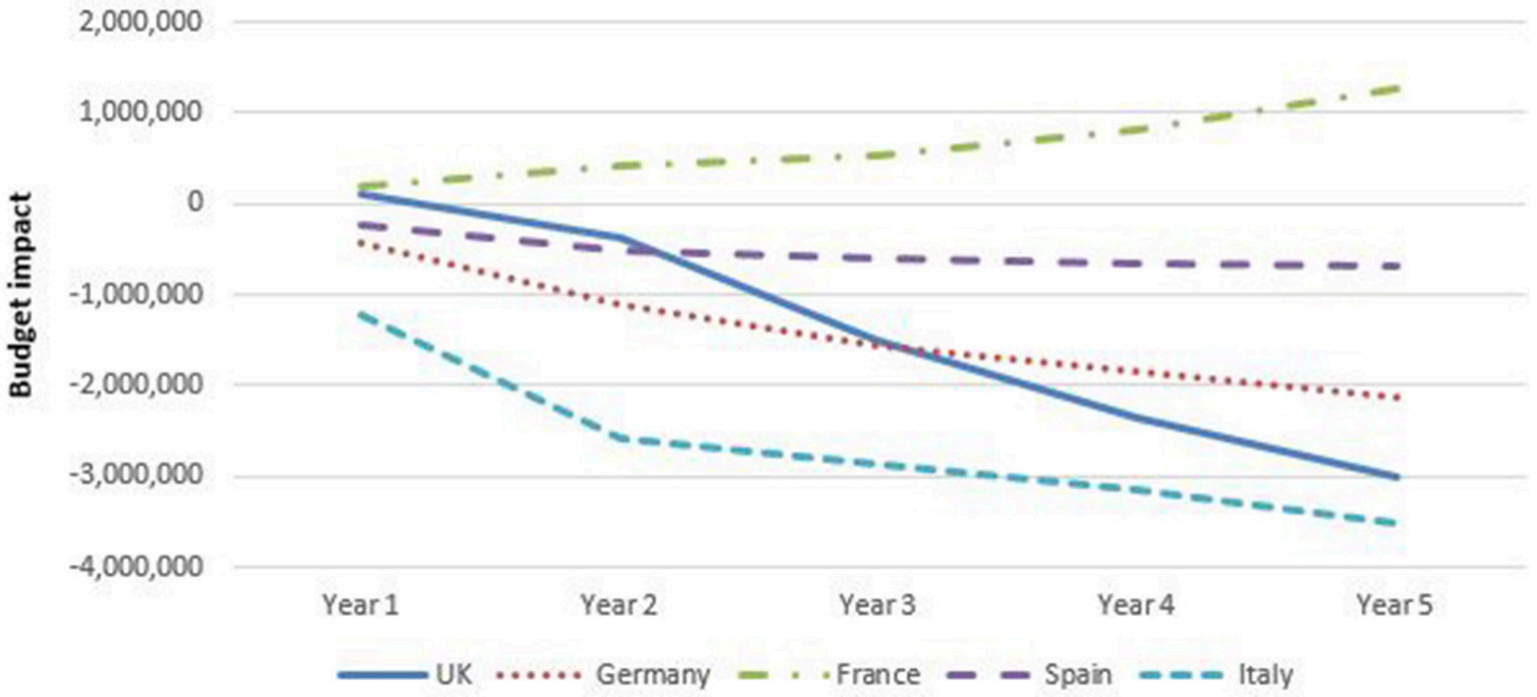
Budget impact of adopting biosimilar infliximab for rheumatology per country, base case.

Figure 6 shows that, except for France, spending for rheumatology (RA plus AS) for year 5 decreased with the introduction of biosimilar infliximab in the base case analyses. In France, the budget increased by adopting biosimilar infliximab. In UK, Germany, Spain, and Italy, savings were obtained from the introduction of biosimilar infliximab in RA in year 5. In France, the budget was virtually unaffected for RA. For AS biosimilar infliximab resulted to a budget savings in Germany, Spain, and Italy, and increased spending in UK and France.

**Figure 7:**
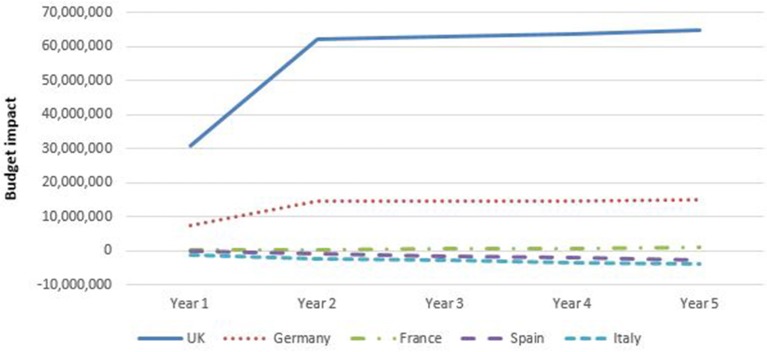
Budget impact of adopting biosimilar infliximab for gastroenterology per country, base case (including vedolizumab for Germany and United Kingdom).

Figure 7 shows that the budget for gastroenterology (CD plus UC) decreased only for Spain and Italy compared to a situation without biosimilar infliximab in the base case analyses. Adopting biosimilar infliximab in UK, Germany and (to a lesser extent) France, showed to increase the budget throughout the 5 year study period. It should be noted that for UK and Germany, these analyses include the adoption of vedulizumab, which had a large effect on the total budget spent on biologicals. These patterns were present for both CD and UC. The increasing budget was particularly prominent in the UK with additional expenditures of €65 million (almost £50 million) in year 5 and €15 million in Germany. Budgets in UK and Germany grew mostly in the first 2 years, after which growth flattened. This was caused by a stabilization in the percentage of patients that shifted to biosimilar infliximab.

The budgetary effect of introducing biosimilar infliximab was larger in gastroenterology than in rheumatology.

### Scenario analyses

Various scenario analyses were performed to examine the influence of specific factors on the budget impact of biosimilar infliximab. The results of the scenario analyses are presented in Table 4. Firstly, the price discount of biosimilar infliximab relative to reference infliximab was varied. If the price of biosimilar infliximab was set at 50% of reference infliximab, adopting biosimilar infliximab decreased the budget in all countries. In rheumatology, budget savings in year 5 varied from €22 million (France) to €81 million (Italy); in gastroenterology savings varied from €12 million (Italy) to almost €250 million (£181 million) in the UK. This was particularly due to the higher proportion of patients that were to be switched from existing reference infliximab to biosimilar infliximab. In addition, the proportion of naïve patients to receive biosimilar infliximab increased. If the price of biosimilar infliximab was set at 75% discount from the reference product (scenario 2), savings were even higher. In rheumatology, savings ranged from €66 million (France) to €289 million (Germany); and from €24 million (Italy) to almost €340 million (£249 million) in UK for gastroenterology.

**Table 4:** Budget impact of adopting biosimilar infliximab: scenario findings (including vedolizumab for Germany and United Kingdom).

	**Year 1**	**Year 2**	**Year 3**	**Year 4**	**Year 5**
**BASE CASE ANALYSES**					
**Rheumatology**					
UK	€ 109,774	€ −361,574	€ −1,516,193	€ −2,357,542	€ −3,015,846
Germany	€ −443,784	€ −1,116,306	€ −1,549,699	€ −1,852,230	€ −2,126,358
France	€ 183,796	€ 426,298	€ 537,628	€ 814,764	€ 1,280,851
Spain	€ −228,679	€ −507,376	€ −603,453	€ −648,025	€ −689,832
Italy	€ −1,225,796	€ −2,595,818	€ −2,870,381	€ −3,149,564	€ −3,526,855
**Gastroenterology**					
UK	€ 30,779,344	€ 61,960,467	€ 62,872,012	€ 63,797,046	€ 64,604,315
Germany	€ 7,420,414	€ 14,728,780	€ 14,681,882	€ 14,752,677	€ 15,020,780
France	€ 104,251	€ 320,745	€ 513,850	€ 670,798	€ 870,614
Spain	€ −283,165	€ −870,727	€ −1,480,663	€ −2,089,845	€ −2,695,510
Italy	€ −1,174,462	€ −2,526,173	€ −2,940,214	€ −3,411,692	€ −3,882,665
**SCENARIO 1: PRICE OF BIOSIMILAR INFLIXIMAB DISCOUNTED FOR 50% OF REFERENCE INFLIXIMAB**					
**Rheumatology**					
UK	€ −15,826,272	€ −53,769,047	€ −57,183,780	€ −60,482,707	€ −63,457,253
Germany	€ −43,782,381	€ −89,078,802	€ −92,058,868	€ −94,918,717	€ −97,649,974
France	€ −9,206,659	€ −18,834,298	€ −19,742,566	€ −20,715,955	€ −21,671,071
Spain	€ −17,360,235	€ −35,112,377	€ −35,975,255	€ −36,910,978	€ −37,821,201
Italy	€ −38,149,588	€ −76,982,705	€ −78,411,624	€ −79,892,237	€ −81,347,278
**Gastroenterology**					
UK	€ −113,570,922	€ −230,027,142	€ −235,708,347	€ −241,213,559	€ −246,548,274
Germany	€ −44,684,033	€ −93,146,186	€ −101,855,520	€ −111,336,722	€ −120,801,286
France	€ −43,764,760	€ −89,661,347	€ −95,017,107	€ −98,776,518	€ −100,307,928
Spain	€ −8,167,878	€ −17,126,717	€ −18,693,461	€ −20,213,632	€ −21,664,256
Italy	€ −3,148,046	€ −6,891,950	€ −8,496,980	€ −10,310,860	€ −11,985,143
**SCENARIO 2: PRICE OF BIOSIMILAR INFLIXIMAB DISCOUNTED FOR 75% OF REFERENCE INFLIXIMAB**					
**Rheumatology**					
UK	€ −56,764,286	€ −156,181,784	€ −162,511,019	€ −168,329,708	€ −173,636,759
Germany	€ −132,789,823	€ −269,178,624	€ −276,530,199	€ −283,256,605	€ −289,091,903
France	€ −28,402,716	€ −58,242,592	€ −61,179,770	€ −63,899,217	€ −66,273,865
Spain	€ −53,320,545	€ −107,952,297	€ −110,680,098	€ −113,124,434	€ −115,128,116
Italy	€ −96,307,582	€ −194,685,762	€ −198,971,950	€ −202,979,944	€ −206,531,593
**Gastroenterology**					
UK	€ −150,569,509	€ −306,944,642	€ −318,021,207	€ −328,753,872	€ −339,153,401
Germany	€ −108,085,499	€ −221,495,452	€ −233,517,906	€ −245,875,301	€ −256,555,585
France	€ −118,182,220	€ −234,622,649	€ −232,142,716	€ −230,935,187	€ −230,239,289
Spain	€ −21,806,068	€ −44,673,440	€ −46,878,688	€ −49,140,862	€ −51,314,227
Italy	€ −8,495,706	€ −18,275,388	€ −20,380,916	€ −22,207,043	€ −24,321,345
**SCENARIO 3: ADDITIONAL CLINICAL TRIAL/OBSERVATIONAL STUDIES EVIDENCE FOR CD AND UC**					
UK	€ 20,363,034	€ 40,691,058	€ 40,641,188	€ 40,687,784	€ 40,787,204
Germany	€ 8,737,610	€ 16,588,897	€ 15,038,121	€ 13,880,180	€ 13,732,601
France	€ 1,422,850	€ 3,226,728	€ 3,682,854	€ 3,897,423	€ 4,173,502
Spain	€ −2,010,138	€ −4,305,483	€ −4,901,324	€ −5,542,733	€ −6,203,693
Italy	€ −3,953,058	€ −8,050,072	€ −8,362,715	€ −8,669,219	€ −8,955,885
**SCENARIO 4: VEDOLIZUMAB AVAILABLE IN ALL EU5 MARKETS FOR CD AND UC**					
UK	€ 30,779,344	€ 61,960,467	€ 62,872,012	€ 63,797,046	€ 64,604,315
Germany	€ 7,420,414	€ 14,728,780	€ 14,681,882	€ 14,752,677	€ 15,020,780
France	€ 29,772,322	€ 60,125,991	€ 61,304,358	€ 62,625,004	€ 64,247,075
Spain	€ 1,988,848	€ 3,739,724	€ 3,398,922	€ 3,269,710	€ 3,223,687
Italy	€ 2,398,784	€ 4,472,121	€ 3,865,715	€ 3,439,909	€ 3,150,509
**SCENARIO 5: ETANERCEPT BIOSIMILAR AVAILABLE FOR RA AND AS**					
UK	€ 109,774	€ −1,444,700	€ −3,927,167	€ −5,254,046	€ −6,403,037
Germany	€ −443,784	€ −6,512,964	€ −13,809,565	€ −17,023,432	€ −20,251,333
France	€ 183,796	€ 168,252	€ −167,228	€ −274,849	€ −192,770
Spain	€ −228,679	€ −2,997,140	€ −6,236,275	€ −7,622,321	€ −9,033,152
Italy	€ −1,225,796	€ −6,966,839	€ −12,859,449	€ −15,679,125	€ −18,678,506
**SCENARIO 6: RITUXIMAB BIOSIMILAR AVAILABLE FOR RA**					
UK	€ 109,774	€ −1,870,365	€ −5,208,772	€ −7,372,658	€ −9,299,708
Germany	€ −443,784	€ −3,556,527	€ −7,531,251	€ −9,992,568	€ −12,340,330
France	€ 183,796	€ 181,972	€ −41,939	€ 55,407	€ 345,741
Spain	€ −228,679	€ −1,193,487	€ −2,120,955	€ −2,453,371	€ −2,777,641
Italy	€ −1,225,796	€ −3,379,697	€ −4,738,065	€ −5,607,527	€ −6,556,249

At the moment of study, evidence for the safety and effectiveness of biosimilar infliximab is currently limited to RA and AS. The third scenario assessed the budget impact if additional evidence would be available for CD and UC. Compared to the base case, savings in Italy and Spain were larger in this scenario. In the UK, the budgetary impact of adopting biosimilar infliximab was smaller than in the base case, but still the budget was higher than the situation without biosimilar infliximab. In France, spending would increase compared to the base case. In Germany, additional evidence would increase the budget in the first years, but lead to savings in years 4 and 5, compared to the base case.

Vedoluzimab was, at the moment the study was performed, only available in UK and Germany (and as such was included in the base case analyses for those countries). Scenario 4 investigated the budget impact if vedoluzimab would also available in the other countries. For all three countries, the introduction of vedoluzimab would lead to an increase in spending compared to the base case. The increase in budget due to vedolizumab was largest in France. This resembles the large budgetary impact of vedolizumab in UK and Germany as shown in the base case analyses.

Scenarios 5 and 6 assessed the budget impact of introducing biosimilar etanercept for RA and AS (scenario 5) and biosimilar rituximab for RA only (scenario 6), in addition to biosimilar infliximab. In all countries, scenarios 5 and 6 led to savings in year 5 compared to a situation without biosimilars (except for France in situation 6). The savings of introducing biosimilar etanercept (scenario 5) were largest in Germany and Italy. Likewise, the savings of introducing biosimilar rituximab (scenario 6) was largest in Germany.

## Discussion

This study assessed the budget impact of adopting biosimilar infliximab for RA, AS, CD, and UC in the EU5, using specialist assumptions derived from a Delphi survey performed in 2015. To the best of the authors' knowledge, this is the first study that calculates the budget impact taking into account the adoption of biosimilar infliximab and the availability of biologic alternatives such as vedolizumab, biosimilar etanercept, and biosimilar rituximab. The study showed that the budgetary impact of introducing biosimilar infliximab varied between countries and between diseases.

In RA, savings would occur in all countries but France, where budget was unaffected by the introduction of biosimilar infliximab. In AS, savings would occur in Germany, Spain, and Italy; in the UK and France budget would increase by introducing biosimilar infliximab. Biosimilar infliximab is used in naïve patients, whereas these patients would have been mostly prescribed reference infliximab if biosimilar infliximab would not be available. As reference infliximab is more expensive, this results in an overall decrease in budget. If savings on budget due to biosimilar infliximab in RA and AS were observed, these were limited in size, as the proportion naïve RA patients to receive biosimilar infliximab was only 5% and the proportion of naïve AS patients to receive biosimilar infliximab was 28% in year 5 (using the current price discounts). For both rheumatologic diseases, (virtually) no patients would switch from other biologics treatment to biosimilar infliximab.

The proportion of IBD patients treated with biosimilar infliximab was much larger than the proportion of RA patients treated with infliximab. For CD and UC, 20% of French naïve patients, 35% of German naïve patients and more than half of the naïve patients in UK, Italy, and Spain received biosimilar infliximab in year 5. Only a small proportion of existing IBD patients would switch, similar to the situation for RA and AS patients. For gastroenterology, adopting biosimilar infliximab would lead to savings in Spain and Italy, in which substantial price reductions were used. In contrast, a substantial increase in the budget in UK and Germany was shown. For these countries, the analyses not only include the adoption of biosimilar infliximab, but also the introduction of vedoluzimab. Vedolizumab is priced higher than existing treatments. Despite the higher price, many CD and UC patients are switched to vedolizumab, reaching a market share of ~15% in year 5 in these two countries. As a result, the budget increased in these two countries.

For France, the budget slightly increased with the introduction of biosimilar infliximab for AS, CD, and UC; the budget for RA was almost equal in the situations with and without biosimilar infliximab. Similarly, budget for AS increased in the UK with the introduction of biosimilar infliximab. Biosimilar infliximab reduced the market share of reference infliximab, but also reduced the market shares of other biologics (etanercept and adalimumab). As total costs for biosimilar infliximab were higher than total costs for etanercept and adalimumab, the budget increased with the introduction of biosimilar infliximab. Theoretically, the introduction of biosimilar infliximab should have no effect on budget in France, as biosimilar infliximab is not superior to reference infliximab in terms of effects or costs, and pateints are therefore not expected to switch. This was observed only for RA. However, for France, the model showed that the budget would increase in AS, CD, and UC. This implies that physicians might have information on additional price discounts of biosimilar infliximab or that they are expecting additional price discounts in the near future.

The proportion of patients that would switch to biosimilar infliximab differed between countries for several reasons. One reason is that price discounts on biosimilar infliximab varied between countries; in France prices for biosimilar infliximab and reference infliximab were equal, whereas in Italy a price discount of 25% applied. The availability of biosimilar infliximab particularly changes market shares for reference infliximab, but also for other available products. To obtain savings, the total cost of biosimilar infliximab would have to be cheaper than other alternatives if provided to naïve patients prior to other alternatives. The increasing budget for AS in the UK and France and for CD and UC in France showed that the current price discounts used in those countries are not sufficient to ensure budget savings. Scenario analyses 1 and 2 show that larger discounts would result in budget savings for all countries. Apparently, physicians are price sensitive, as larger discounts would lead to higher proportions of patients to receive biosimilar infliximab. However, their price sensitivity is limited, as the majority of patients would be switched only if large discounts are in place. Physicians' price sensitivity is also related to national policies, which can stimulate the use of biosimilars in various ways. For instance, specific regulation is installed in Norway to increase the uptake of biosimilars, amongst which biosimilar infliximab, and reduce biosimilar prices substantially (Jahnsen and Kaasen Jorgensen, 2017).

Other reasons include physicians' beliefs about the comparability of biosimilars to reference products. There are strict regulations about comparability of biosimilars (Simoens et al., 2012), but still not all physicians in our sample believed that biosimilar infliximab was perfectly comparable to the reference product. As a result, they might be less inclined to switch patients from reference product to biosimilar.

### Limitations

The study was based on input from a Delphi survey conducted in 2015. Since then, several developments have taken place that need to be taken into account when interpreting the results from our study. In addition to Inflectra® and Remsima®, another biosimilar infliximab (marketed as Flixabi®) is now becoming available in European countries. Also, the uptake of biosimilar products since then has exceeded expectations, which can be attributed to various reasons. Firstly, actual discounts on prices have been larger than presented to the respondents of the Delphi panel. Secondly, specialists are more willing to prescribe biosimilars. This was recently found in a survey among IBD specialists, performed by ECCO (Danese et al., 2016). The specialists state that they have less concerns and more confidence in the use of biosimilars than was previously the case. National rheumatology guidelines in the UK, Germany, and Belgium include guidance to prescribe biosimilar products (Dörner et al., 2016). Another development that has been observed since the Delphi survey was conducted, is that other biosimilars have entered the market, such as biosimilar etanercept. Scenario five showed that the introduction of biosimilar etanercept would lead to budget savings. Many more biosimilars are in the pipeline for RA and IBD (Dörner and Kay, 2015; Dörner et al., 2016). When these will reach the market, this could lead to competition and pressure on prices of biosimilars and innovator products, as we see already happening. As a result, a larger proportion of patients are expected to be treated with biosimilars, as was shown in scenarios 1 and 2, leading to larger savings.

The coefficient of variation was used to assess the dispersion of respondents' answers. Differences in answers were particularly prominent with respect to the expected percentage of naïve patients to receive biosimilar infliximab and the percentage of existing patients to switch from other treatments to biosimilar infliximab. For this reason, median values were used in the study (rather than mean values), to prevent that the effect of outliers on the values used in the model would be too big. For rheumatology, although at least one respondent per country was included in the sample, the number of respondents was deemed too small to calculate country-specific values. Therefore, answers for the five countries were aggregated to one EU5 average. Epidemiological data and input parameters regarding costs used in the model were country-specific. Whether responses from experts in the Delphi survey were representative of their country could not be assess. However, variation between experts participating in the study was limited, suggesting that their answers were representative.

Price changes for existing therapies were neglected in the study. In reality, prices of existing products might be lowered if competition increases with the entry of other (biosimilar) products. Furthermore, the study did not take into account possible market access agreements e.g., discounts on list prices or other risk sharing agreements. As a result, the current estimates of budget savings might be overestimated and increases in spending might be underestimated.

We assumed that no vial sharing occurred in order to ensure comparability of resource use and drug acquisition costs between countries, although in practice vial sharing may occur. However, this practice is variable and likely to differ between countries and between hospitals within a country. As data on vial sharing are not widely available and may not be generalizable, we preferred to assume that no vial sharing occurred in our analysis.

Vedolizumab was included in the base case as a treatment option for CD and UC in the UK and Germany to adequately reflect the available treatments in these countries. However, this complicates the assessment of the impact of biosimilar infliximab on the budget in these countries, as the impact on the budget of biosimilar infliximab and vedolizumab could not be disentangled. If the price of vedolizumab was set equal to the price of biosimilar infliximab, savings in the budget were shown for the UK and Germany, resembling observations for Spain and Italy. Likewise, the budgets in France, Spain, and Italy, increased when vedolizumab was assumed to be available in these countries as well, as shown in scenario 4.

### Other studies

Various studies have investigated the impact of biosimilars on healthcare budgets. Our results deviate from earlier, assumption-based predictions on the impact of biosimilar infliximab on healthcare budgets that projected budget savings for various European countries in both rheumatology and gastroenterology (McCarthy et al., 2013; Brodszky et al., 2014; Jha et al., 2014; Kim et al., 2014). The approach in these studies particularly varied from our methodology using an (arbitrary) value for the price reductions of biosimilar infliximab, instead of using discounts as provided in national list prices. In the scenario analyses, we showed that our model also projects savings when larger discounts were used. Importantly, our results for UK and Germany also deviate because of the inclusion of vedolizumab in our study, associated with a large increase in the budget.

Scenario 5 in our study, investigating the impact of the introduction of a biosimilar for etanercept, resembled the findings of an decreasing budget also found in an earlier study investigating budget impact in EU5 countries (Ruff et al., 2015).

### Implications

The number of biosimilars in the pipeline to replace the biologic treatmens in rheumatology and gastroenterology is very large (Dörner and Kay, 2015; Dörner et al., 2016), and underlines both the importance of this study and the opportunity for policy makers to obtain reductions in healthcare budgets. When these biosimilar products will reach the market, it is likely that this will result in downward pressure on prices of all biological treatments, leading to discounts that are larger than the ones used in this study. Besides, list prices, as used in the base case analyses, probably do not reflect actual price levels of biosimilar infliximab, as confidential price agreements were not included in the analyses. Although further research, using updated prices and treatment options and including (confidential) price agreements, is needed to establish the actual budget impact of adopting biosimilar infliximab, scenario 1, using a price discount of 50% on biosimilar infliximab, is in our opinion likely to reflect the actual budget impact of biosimilar infliximab better than the base case analyses.

The purpose of introducing biosimilars is to constrain the growing financial impact of expensive biologic treatments and/or to expand access to healthcare. In our study, the introduction of biosimilar infliximab would lead to increasing budgets in some countries instead and would only lead to modest savings in other countries. In scenario analyses 1 and 2, we have shown that when price discounts are large enough physicians will be more prone to change their prescribing behavior and then budgetary savings will occur. Policy makers should therefore focus on substantial price reductions for biosimilar products. Furthermore, our study showed that the proportion of patients to be switched from reference infliximab to biosimilar infliximab is limited (0–10%). Policy makers can increase budgetary savings by stimulating transition from reference biologics to biosimilar products. In this respect, policy makers, and prescribers should be made aware of the fact that biosimilar products are as safe and effective as reference products, but that they come at a lower price.

## Author contributions

SS, IH, AV, and JS provided substantial contributions to the conception and design of the work. JS conducted the Delphi data acquisition analysis and modified and updated the basic budget impact model. TK reviewed and validated the budget impact model. TK and JS jointly wrote a first draft of the manuscript and SS, IH, and AV revised critically for important intellectual content. All authors approved the final version to be published and are all accountable for all aspects of the work in ensuring that questions related to the accuracy or integrity of any part of the work are appropriately investigated and resolved.

### Conflict of interest statement

SS, IH, and AV are the founders of the KU Leuven Fund on Market Analysis of Biologics and Biosimilars following Loss of Exclusivity (MABEL). SS and IH are involved in a stakeholder roundtable on biosimilars sponsored by Amgen and MSD. SS has participated in an advisory board meeting on biosimilars for Pfizer. He is also in the process of conducting a systematic review of budget impact models for biosimilars in chronic inflammatory diseases in collaboration with Pfizer, for which no compensation is paid. AV is involved in consulting, advisory work, and speaking engagements for a number of companies, a.o. AbbVie, Amgen, Biogen, Boehringer Ingelheim, EGA, Pfizer/Hospira, Mundipharma, Roche, Sandoz. He has no personal benefit from these activities; any compensation is paid to his employer. The authors declare that the research was conducted in the absence of any commercial or financial relationships that could be construed as a potential conflict of interest

## Footnotes

^1^Data Market. *HICP (2005* = *100) - Annual Data (Average Index and Rate of Change)*. Available online at: https://datamarket.com/data/set/18qu/hicp-2005-100-annual-data-average-index-and-rate-of-change#!ds=18qu!mh6=2:mh7=1m:6ggm=1.2.3.j.5.6.8.b.h.i.7.d.k.m.l.n.q.o.p.r.s.t.u.v.w.z.y.e.x.4.10.11&display=choropleth&map=europe&classifier=natural&numclasses=5 (Accessed January 05, 2015).
